# Broad spectrum in vitro microbicidal activity of benzoyl peroxide against microorganisms related to cutaneous diseases

**DOI:** 10.1111/1346-8138.15739

**Published:** 2020-12-28

**Authors:** Kazuaki Okamoto, Shoji Kanayama, Fumiaki Ikeda, Koki Fujikawa, Shiori Fujiwara, Naoki Nozawa, Sachi Mori, Tatsumi Matsumoto, Naoki Hayashi, Masataka Oda

**Affiliations:** ^1^ Information Systems Department Maruho Co., Ltd. Osaka Japan; ^2^ Department of Microbiology and Infection Control Science Kyoto Pharmaceutical University Kyoto Japan; ^3^ Drug Development Research Laboratories Kyoto R&D Center Maruho Co., Ltd. Kyoto Japan; ^4^ Drug Discovery Research Department Kyoto R&D Center Maruho Co., Ltd. Kyoto Japan

**Keywords:** Benzoyl peroxide, *Cutibacterium*, *Malassezia*, microbicidal, *Staphylococcus*

## Abstract

The in vitro microbicidal activity of benzoyl peroxide against *Cutibacterium acnes*, *Staphylococcus aureus*, *Staphylococcus epidermidis*, *Escherichia coli*, *Pseudomonas aeruginosa*, *Candida albicans*, *Malassezia furfur*, *Malassezia restricta,* and *Malassezia globosa* was investigated. These strains were incubated for 1 h in the presence of 0.25, 0.5, 1, or 2 mmol/L benzoyl peroxide in phosphate buffered saline supplemented with 0.1% glycerol and 2% Tween 80. After exposure to benzoyl peroxide, counts of viable Gram‐positive bacteria and fungi were markedly decreased, whereas counts of Gram‐negative bacteria were unchanged. Transmission electron microscopy images showed a decrease in electron density and the destruction of *C. acnes* and *M. restricta* cell walls after exposure to 2 mmol/L benzoyl peroxide. In conclusion, this study showed that benzoyl peroxide has a potent and rapid microbicidal activity against Gram‐positive bacteria and fungi that are associated with various cutaneous diseases. This suggests that the direct destruction of bacterial cell walls by benzoyl peroxide is an essential mechanism of its rapid and potent microbicidal activity against microorganisms.

## 1 INTRODUCTION

Benzoyl peroxide (BPO) is widely used as a topical drug for acne vulgaris,^1^ and has keratolytic/comedolytic, anti‐inflammatory effects^1^ and antimicrobial activity against *Cutibacterium acnes*.^2^, ^3^ We also previously demonstrated the rapid and potent bactericidal activity of BPO against *C. acnes*.^4^ BPO is rapidly degraded to benzoic acid to generate free radicals,^5^ and it is thought to cause damage to the bacterial cell walls.^6^ Therefore, BPO may be effective against microorganisms other than *C. acnes*. Akaza et al. reported the possibility that not only *C. acnes* but also other cutaneous resident microorganisms such as *Staphylococcus* species and *Malassezia* species are related to acne.^7^ In this study, we investigated the in vitro microbicidal activity of BPO against various bacteria and fungi.

## 2 METHODS

Microorganisms used in this study were *C. acnes* ATCC11827, *Staphylococcus aureus* ATCC29213, *S. epidermidis* ATCC12228, *Escherichia coli* ATCC25922, *Pseudomonas aeruginosa* ATCC27853, *Candida albicans* ATCC90028, *Malassezia furfur* ATCC14521, *M. restricta* ATCC MYA‐4611, and *M. globosa* ATCC MYA‐4612. *C. acnes* was cultured on Anaero Columbia agar supplemented with 5% rabbit blood (Nippon Becton Dickinson Company Ltd., Tokyo, Japan) for 72 h at 35°C under anaerobic conditions using an AnaeroPack system (Mitsubishi Gas Chemical Company Inc, Tokyo, Japan). *S. aureus*, *S. epidermidis*, *E. coli* and *P. aeruginosa* were cultured on Mueller‐Hinton agar (Nippon Becton Dickinson Company Ltd.) for 24 h at 35°C under aerobic conditions. *C. albicans* was cultured on Sabouraud agar (Nippon Becton Dickinson Company Ltd.) for 24 h at 32°C under aerobic conditions. *M. furfur* and *M. globosa* were cultured on modified Leeming and Notman agar^8^ for 3 or 7 days at 32°C under aerobic conditions and *M. restricta* was cultured on modified Leeming and Notman agar 5–7 days at 32°C under microaerobic conditions using an AnaeroPack system (Mitsubishi Gas Chemical Company Inc.). BPO was purchased from Sigma‐Aldrich (St. Louis, MO, USA).

The microbicidal activity of BPO was measured by an in vitro assay method reported previously.^4^ Bacteria and fungi were cultured using the above conditions and suspended at approximately 10^6^ colony forming units (CFU)/mL in phosphate buffered saline (PBS) supplemented with 0.1% glycerol and 2% Tween 80. BPO dissolved in dimethyl sulfoxide (DMSO) was added to the bacterial or fungal suspensions at a final concentration ranging from 0.25 to 2 mmol/L (final concentration of DMSO was 3%). After aerobic incubation for 1 h at 35°C, 100 µL of the bacterial or fungal suspensions was removed, serially diluted, plated onto agar, and cultured using the above growth conditions. The number of colonies on agar plates was counted (detection limit, 1.60 log CFU/mL). To investigate the influence of the outer membrane on the bactericidal activity of BPO against *E. coli* and *P. aeruginosa*, these strains were preincubated aerobically in PBS with 100 mmol/L ethylenediaminetetraacetic acid (EDTA) for 1.5 h at 35°C. After washing the cells twice with PBS, the bactericidal activity of BPO was measured. Each assay was repeated in triplicate. Statistical analyses were performed by Dunnett's multiple comparison test and Student's t‐test using EXSUS ver. 8.0.0 (CAC Croit Corporation, Tokyo, Japan). *P*‐values less than 0.05 were considered significant.

The effect of BPO on cell morphology was evaluated by transmission electron microscopy (TEM). These strains were exposed to 2 mmol/L BPO for 1 h at 35°C under aerobic conditions. The cells were fixed with 2.5% glutaraldehyde in 0.1 mol/L cacodylate buffer at 4°C overnight. After washing several times with 0.1 mol/L cacodylate buffer, cells were post‐fixed with 1.5% potassium permanganate in water at 4°C for 16 h. Following acetone dehydration, samples were embedded in plain resin (Nisshin EM Co., Tokyo, Japan). Ultrathin sections were observed and photographed with a transmission electron microscope (H‐7600, Hitachi, Tokyo, Japan) operated at 200 kV.

## 3 RESULTS

**Figure 1:**
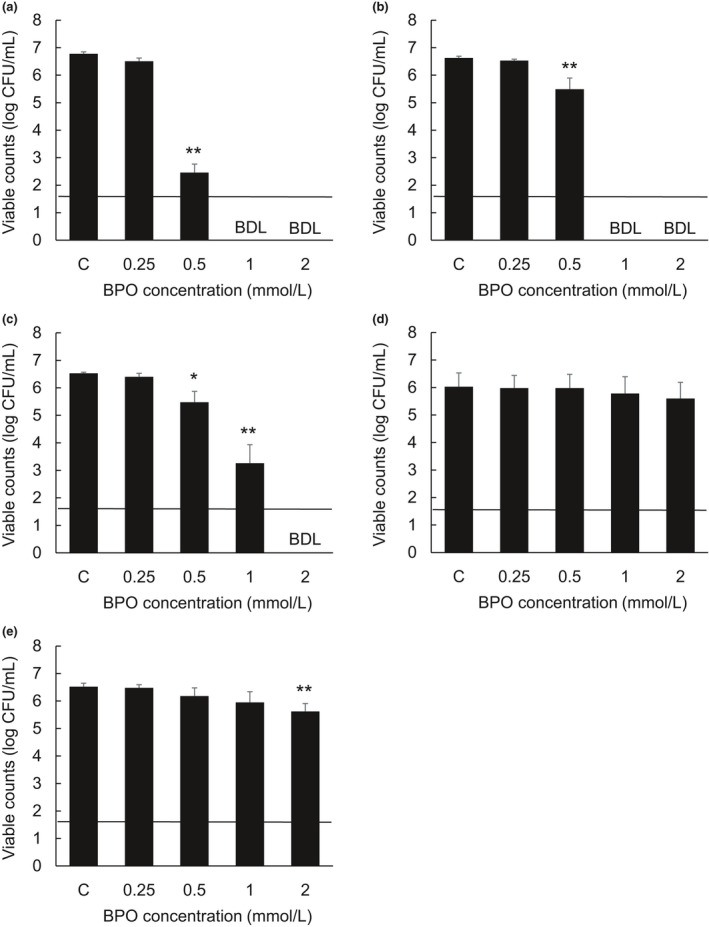
Microbicidal activity of benzoyl peroxide against various bacterial strains. *Cutibacterium acne*s ATCC11827 (a), *Staphylococcus aureus* ATCC29213 (b), *Staphylococcus epidermidis* ATCC12228 (c), *Escherichia coli* ATCC25922 (d), and *Pseudomonas aeruginosa* ATCC27853 (e) were incubated for 1 h with 0.25, 0.5, 1, or 2m mol/L benzoyl peroxide (BPO). After incubation, the bacterial suspensions were collected and plated onto agar plates to calculate viable counts. Data indicate the mean ± standard deviation of three repeated experiments. CFU, colony forming unit; C, control; BDL, below the detection limit (<1.60 log CFU/mL). *P < 0.05, **P < 0.01 compared with the control group (Dunnett's multiple comparison test, 2‐sided)

Figure 1 shows the microbicidal activity of BPO against five species of bacteria. The number of colonies of *C. acnes*, *S. aureus*, and *S. epidermidis* were decreased significantly by exposure to 0.5, 1, and 2 mmol/L BPO. However, the number of colonies of *P. aeruginosa* was only decreased when exposed to 2 mmol/L BPO. Moreover, the number of colonies of *E. coli* was not decreased by exposure to any concentration of BPO, including 2 mmol/L. The number of EDTA‐pretreated *E. coli* and *P. aeruginosa* colonies was decreased significantly by exposure to 2 mmol/L BPO for 1 h ([Link], [Link], [Link]).

**Figure 2:**
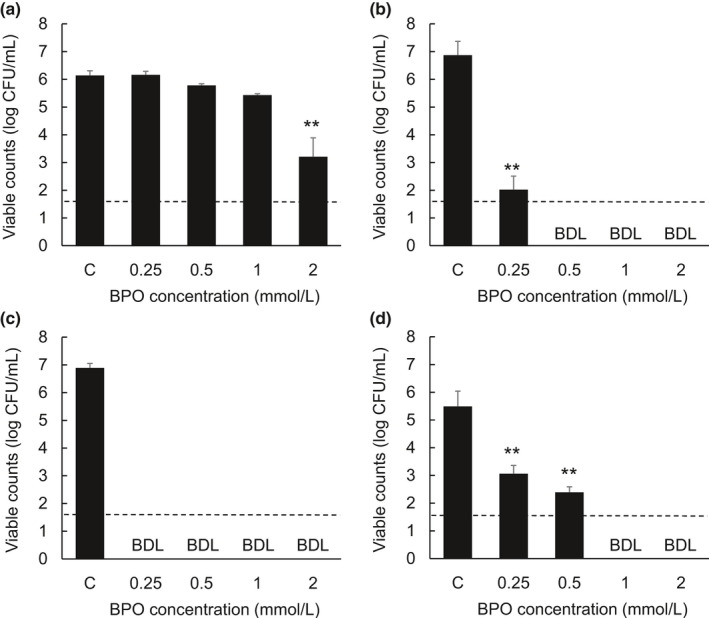
Microbicidal activity of benzoyl peroxide against various fungal strains. *Cutibacterium albicans* ATCC90028 (a), *Malassezia furfur* ATCC14521 (b), *Malassezia restricta* ATCC MYA‐4611 (c), and *Malassezia globosa* ATCC MYA‐4612 (d) were incubated for 1 h with 0.25, 0.5, 1, or 2 mmol/L benzoyl peroxide (BPO). After incubation, the fungal suspensions were collected and plated onto agar plates to calculate the viable counts. Data indicate the mean ± standard deviation of three repeated experiments. CFU, colony forming unit; C, control; BDL, below the detection limit (<1.60 log CFU/mL). **P < 0.01, compared with the control group (Dunnett's multiple comparison test, 2‐sided)

Figure 2 shows the microbicidal activity of BPO against four species of fungi. The number of colonies of *C. albicans* was only decreased significantly when exposed to 2 mmol/L BPO. In contrast, the number of colonies of *M. furfur*, *M. restricta*, and *M. globosa* was decreased significantly when exposed to 0.25, 0.5, 1, and 2m mol/L BPO.

**Figure 3:**
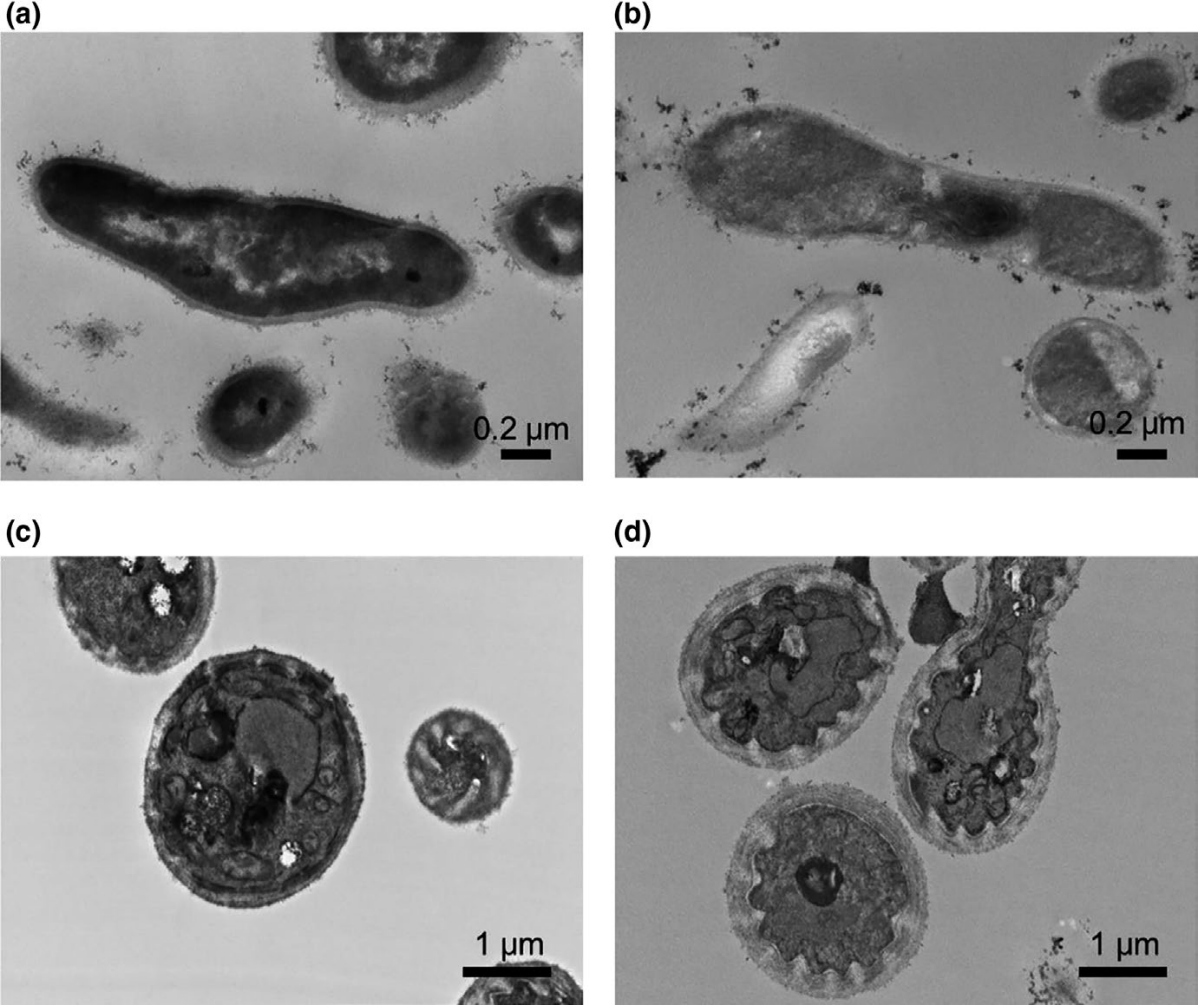
Morphological changes of *Cutibacterium acnes* and *Malassezia restricta* after exposure to benzoyl peroxide. *C. acnes* and *M. restricta* were exposed to 2 mmol/L benzoyl peroxide (BPO) for 1 h and morphological changes were observed by transmission electron microscopy. Control *C. acnes* (a), 2 mmol/L BPO‐treated *C. acnes* (magnification: ×60 000) (b), control *M. restricta* (c), and 2 mmol/L BPO‐treated *M. restricta* (magnification, ×20 000) (d)

Figure 3 shows the influence of BPO on the cell morphology of *C. acnes* and *M. restricta* by TEM *C. acnes* and *M. restricta* after exposure of BPO (Figure 3B,D) showed a rough and blurry cell wall. In *C. acnes*, cell wall was disrupted partly, cytoplasmic materials were released to the extracellular (Figure 3B). In *M. restricta*, cell wall was expanded and electron density of it was decreased obviously (Figure 3D). These findings suggest that BPO directly damaged the cell wall of these strains, causing their death.

## 4 DISCUSSION

Benzoyl peroxide had a potent and rapid microbicidal activity against Gram‐positive bacteria and fungi within 1 h. That is, significant decrease of viable counts of *C. acnes*, *S. aureus*, *S. epidermidis* and *Malassezia* species was observed at 15, 30 and 60 min after exposure of 2 mmol/L BPO ([Link], [Link], [Link]). Especially, *Malassezia* species were more sensitive to BPO than other bacteria. The cell wall of *Malassezia* is surrounded by a lipid‐rich outer layer.^9^ BPO is a highly lipophilic compound^1^ and therefore might more easily penetrate the cell wall of *Malassezia* than bacterial cell walls. It has been reported that *Malassezia* species, especially *M. globosa* and *M. restricta* are involved in the pathogenesis of acne vulgaris.^7^ The microbicidal activity of BPO against those *Malassezia* species may contribute to the effectiveness of the acne treatment. Various *Malassezia* species present on human skin as commensals are also associated with multiple skin diseases including pityriasis versicolor, *Malassezia* folliculitis, seborrheic dermatitis, atopic dermatitis, and psoriasis. A previous study reported that *M. globosa* and *M. restricta* were the predominating species isolated from patients with these diseases.^10^ However, BPO may not be used for these diseases, especially atopic dermatitis or psoriasis, because skin irritation, scales or erythema may occur during the use of this drug.

Benzoyl peroxide had a weaker bactericidal activity against Gram‐negative bacteria within 1 h. Gram‐negative bacteria have an outer membrane that functions as a permeability barrier against macromolecules and hydrophilic substances.^11^, ^12^ EDTA is a chelating agent that damages the outer membrane of Gram‐negative bacteria by forming a chelate complex with a divalent metal ion such as Ca^2+^ or Mg^2+^ present in the outer membrane.^11^, ^12^ BPO showed potent bactericidal activity against EDTA‐pretreated *E. coli and P. aeruginosa*. These results suggest that the bactericidal activity of BPO against *E. coli* and *P. aeruginosa* was blocked by their outer membrane.

In TEM observation, decreased electron density and the partial destruction of cell walls in *C. acnes* and *M. restricta* were observed after exposure to BPO. These morphological changes were similar to those in *E. coli* and *S. aureus* treated with short time ultrasound which is reported to cause a direct damage to the cell walls in a previous study.^13^ These findings suggest that BPO directly damaged the cell wall of these strains, causing their death.

In conclusion, this study showed that BPO has potent and rapid microbicidal activity against microorganisms that are associated with cutaneous diseases and the direct destruction of bacterial cell walls by BPO is an essential mechanism of its rapid and potent microbicidal activity against microorganisms.

## CONFLICT OF INTEREST

Kazuaki Okamoto, Shoji Kanayama, Fumiaki Ikeda, Naoki Hayashi, Koki Fujikawa, Shiori Fujiwara, Naoki Nozawa, Sachi Mori, Tatsumi Matsumoto are employees of Maruho Co., Ltd.. Masataka Oda is collaborator of Maruho Co., Ltd.

## Supporting information


**Figure S1**

**Table S1**

**Table S2**

## References

[1] Sagransky M , Yentzer BA , Feldman SR . Benzoyl peroxide: a review of its current use in the treatment of acne vulgaris. Expert Opin Pharmacother. 2009;10:2555–62. 19761357 10.1517/14656560903277228

[2] Decker LC , Deuel DM , Sedlock DM . Role of lipids in augmenting the antibacterial activity of benzoyl peroxide against *Propionibacterium acnes* . Antimicrob Agents Chemother. 1989;33:326–30. 2729927 10.1128/aac.33.3.326 PMC171487

[3] Nakatsuji T , Kao MC , Fang J‐Y , Zouboulis CC , Zhang L , Gallo RL , et al. Antimicrobial property of lauric acid against *Propionibacterium acne*s: its therapeutic potential for inflammatory acne vulgaris. J Invest Dermatol. 2009;129:2480–8. 19387482 10.1038/jid.2009.93 PMC2772209

[4] Okamoto K , Ikeda F , Kanayama S , Nakajima A , Matsumoto T , Ishii R , et al. In vitro antimicrobial activity of benzoyl peroxide against *Propionibacterium acne*s assessed by a novel susceptibility testing method. J Infect Chemother. 2016;22:426–9. 26806150 10.1016/j.jiac.2015.12.010

[5] Taylor GA , Shalita AR . Benzoyl peroxide‐based combination therapies for acne vulgaris: a comparative review. Am J Clin Dermatol. 2004;5:261–5. 15301572 10.2165/00128071-200405040-00005

[6] Friedman AJ , Phan J , Schairer DO , Champer J , Qin M , Pirouz A , et al. Antimicrobial and anti‐inflammatory activity of chitosan‐alginate nanoparticles: a targeted therapy for cutaneous pathogens. J Invest Dermatol. 2013;133:1231–9. 23190896 10.1038/jid.2012.399 PMC3631294

[7] Akaza N , Akamatsu H , Numata S , Yamada S , Yagami A , Nakata S , et al. Microorganisms inhabiting follicular contents of facial acne are not only *Propionibacterium* but also *Malassezia spp* . J Dermatol. 2016;43:906–11. 26705192 10.1111/1346-8138.13245

[8] Leeming JP , Notman FH . Improved methods for isolation and enumeration of *Malassezia furfur* from human skin. J Clin Microbiol. 1987;25:2017–9. 3667925 10.1128/jcm.25.10.2017-2019.1987 PMC269393

[9] Mittag H . Fine structural investigation of *Malassezia furfur*. II. The envelope of the yeast cells. Mycoses. 1995;38:13–21. 7637677 10.1111/j.1439-0507.1995.tb00003.x

[10] Prohic A , Jovovic Sadikovic T , Krupalija‐Fazlic M , Kuskunovic‐Vlahovljak S *Malassezia* species in healthy skin and in dermatological conditions. Int J Dermatol. 2016;55:494–504. 26710919 10.1111/ijd.13116

[11] Alakomi HL , Paananen A , Suihko ML , Helander IM , Saarela M . Weakening effect of cell permeabilizers on gram‐negative bacteria causing biodeterioration. Appl Environ Microbiol. 2006;72:4695–703. 16820461 10.1128/AEM.00142-06 PMC1489302

[12] Nikaido H . Molecular basis of bacterial outer membrane permeability revisited. Microbiol Mol Biol Rev. 2003;67:593–656. 14665678 10.1128/MMBR.67.4.593-656.2003 PMC309051

[13] Li J , Ahn J , Liu D , Chen S , Ye X , Ding T . Evaluation of ultrasound‐induced damage to *Escherichia coli* and *Staphylococcus aureus* by flow cytometry and transmission electron microscopy. Appl Environ Microbiol. 2016;82:1828–37. 26746712 10.1128/AEM.03080-15 PMC4784048

