# Supplementary material for: Broad spectrum in vitro microbicidal activity of benzoyl peroxide against microorganisms related to cutaneous diseases
Source: J Dermatol. 2020 Dec 28;48(4):551–5. doi: 10.1111/1346-8138.15739 (PMC8048985; doi:10.1111/1346-8138.15739)
Supplement: Supplementary file 1 — Figure S1 [file JDE-48-551-s001.docx]

**Supporting information**

Supplementary Figure 1. Effect of ethylenediaminetetraacetic acid on the bactericidal activity of benzoyl peroxide against Gram-negative bacteria.


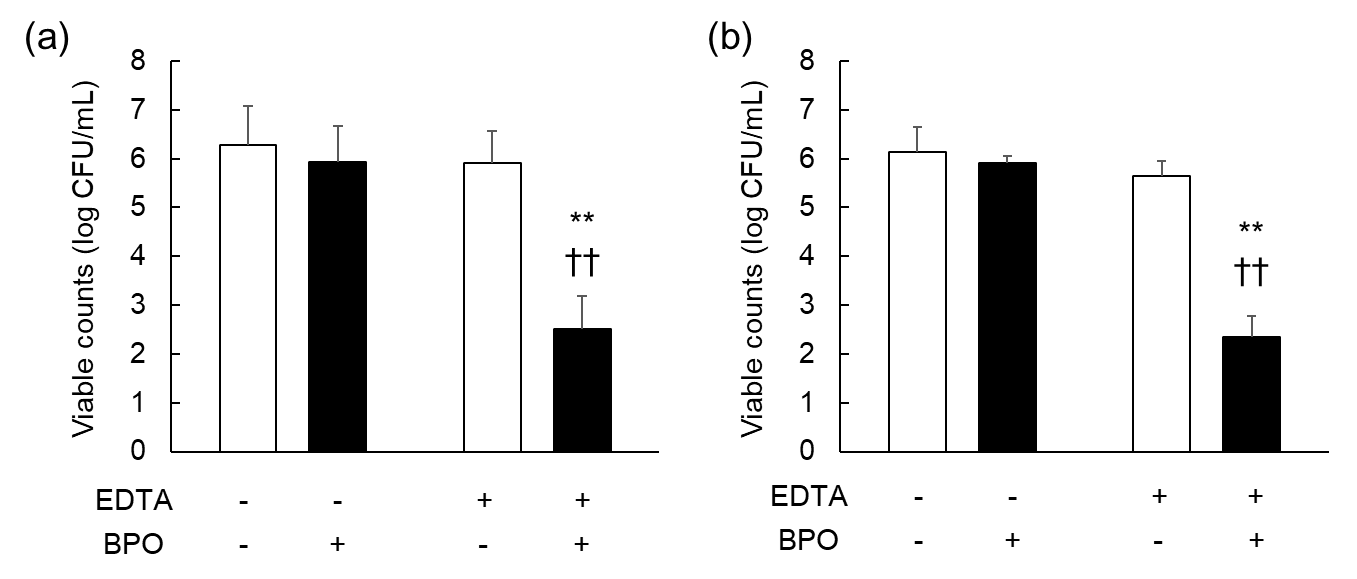


*E. coli* ATCC25922 (a) and *P. aeruginosa* ATCC27853 (b) were pretreated with 100 mmol/L ethylenediaminetetraacetic acid (EDTA) for 90 minutes prior to benzoyl peroxide (BPO) exposure. After washing with phosphate buffered saline, bacteria were incubated for 60 minutes with 2 mmol/L BPO. The bacterial suspension was collected and plated onto agar plates to calculate the viable bacterial count. Data indicates the mean ± standard deviation of three repeated experiments. CFU: colony forming unit.

***P*<0.01, compared with the EDTA (-) and BPO (+) groups (Student’s *t*-test, 2-sided).

^††^*P*<0.01, compared with the EDTA (+) and BPO (-) groups (Student’s *t*-test, 2-sided).
