# Supplementary material for: Broad spectrum in vitro microbicidal activity of benzoyl peroxide against microorganisms related to cutaneous diseases
Source: J Dermatol. 2020 Dec 28;48(4):551–5. doi: 10.1111/1346-8138.15739 (PMC8048985; doi:10.1111/1346-8138.15739)
Supplement: Supplementary file 2 — Table S1 [file JDE-48-551-s003.doc]

**Supporting information**

Supplementary Table 1. The impact of BPO on viable counts of bacteria in each time point.

| Bacteria | Time (m) | Viable counts ^a)^ (log CFU/mL) | | | | |
| --- | --- | --- | --- | --- | --- | --- |
|  |  | Control | 0.25 mmol/L | 0.5 mmol/L | 1 mmol/L | 2 mmol/L |
| *C. acnes* ATCC11827 | 0 | 6.78 | 6.71 | 6.72 | 6.78 | 6.77 |
|  | 15 | n.t. ^b)^ | 6.81 | 6.69 | 5.55 | 4.41 |
|  | 30 | n.t. ^b)^ | 6.66 | 5.06 | 3.32 | BDL ^c)^ |
|  | 60 | 6.78 | 6.51 | 2.46 | BDL ^c)^ | BDL ^c)^ |
| *S. aureus* ATCC29213 | 0 | 6.62 | 6.63 | 6.64 | 6.66 | 6.64 |
|  | 15 | n.t. ^b)^ | 6.61 | 6.61 | 6.52 | 4.78 |
|  | 30 | n.t. ^b)^ | 6.64 | 6.36 | 5.18 | BDL ^c)^ |
|  | 60 | 6.63 | 6.53 | 5.49 | BDL ^c)^ | BDL ^c)^ |
| *S. epidermidis* ATCC12228 | 0 | 6.55 | 6.52 | 6.53 | 6.56 | 6.54 |
|  | 15 | n.t. ^b)^ | 6.47 | 6.55 | 6.52 | 5.85 |
|  | 30 | n.t. ^b)^ | 6.56 | 6.30 | 5.52 | 3.90 |
|  | 60 | 6.53 | 6.40 | 5.48 | 3.26 | BDL ^c)^ |
| *E. coli* ATCC25922 | 0 | 5.98 | 6.01 | 6.02 | 6.02 | 6.05 |
|  | 15 | n.t. ^b)^ | 6.04 | 6.02 | 6.02 | 5.89 |
|  | 30 | n.t. ^b)^ | 5.99 | 5.94 | 5.83 | 5.78 |
|  | 60 | 6.03 | 5.98 | 5.98 | 5.78 | 5.60 |
| *P. aeruginosa* ATCC27853 | 0 | 6.53 | 6.56 | 6.56 | 6.57 | 6.56 |
|  | 15 | n.t. ^b)^ | 6.56 | 6.51 | 6.42 | 6.20 |
|  | 30 | n.t. ^b)^ | 6.51 | 6.43 | 6.18 | 5.81 |
|  | 60 | 6.52 | 6.48 | 6.18 | 5.95 | 5.62 |

^a)^ Bacteria was incubated for 15, 30 and 60 minutes with 0.25, 0.5, 1 or 2 mmol/L benzoyl peroxide (BPO). After incubation, the bacterial suspensions were collected and plated onto agar plates to calculate viable counts. Data indicate the mean of three repeated experiments. CFU: colony forming unit.

^b)^ Not tested.

^c)^ BDL: below the detection limit (<1.60 log CFU/mL).
