# Supplementary material for: Broad spectrum in vitro microbicidal activity of benzoyl peroxide against microorganisms related to cutaneous diseases
Source: J Dermatol. 2020 Dec 28;48(4):551–5. doi: 10.1111/1346-8138.15739 (PMC8048985; doi:10.1111/1346-8138.15739)
Supplement: Supplementary file 3 — Table S2 [file JDE-48-551-s002.doc]

**Supporting information**

Supplementary Table 2. The impact of BPO on viable counts of fungi in each time point.

| Fungi | Time (m) | Viable counts ^a)^ (log CFU/mL) | | | | |
| --- | --- | --- | --- | --- | --- | --- |
|  |  | Control | 0.25 mmol/L | 0.5 mmol/L | 1 mmol/L | 2 mmol/L |
| *C. albicans* ATCC90028 | 0 | 6.15 | 6.21 | 6.14 | 6.19 | 6.19 |
|  | 15 | n.t. ^b)^ | 6.16 | 6.18 | 6.13 | 5.86 |
|  | 30 | n.t. ^b)^ | 6.10 | 6.05 | 5.87 | 5.35 |
|  | 60 | 6.14 | 6.16 | 5.78 | 5.43 | 3.21 |
| *M. furfur* ATCC14521 | 0 | 6.85 | 6.75 | 6.81 | 6.79 | 6.79 |
|  | 15 | n.t. ^b)^ | 3.79 | 2.48 | BDL ^c)^ | BDL ^c)^ |
|  | 30 | n.t. ^b)^ | 2.68 | BDL ^c)^ | BDL ^c)^ | BDL ^c)^ |
|  | 60 | 6.87 | 2.02 | BDL ^c)^ | BDL ^c)^ | BDL ^c)^ |
| *M. restricta* ATCC MYA-4611 | 0 | 6.86 | 6.79 | 6.87 | 6.82 | 6.83 |
|  | 15 | n.t. ^b)^ | 3.85 | 2.64 | 2.35 | BDL ^c)^ |
|  | 30 | n.t. ^b)^ | BDL ^c)^ | BDL ^c)^ | BDL ^c)^ | BDL ^c)^ |
|  | 60 | 6.03 | BDL ^c)^ | BDL ^c)^ | BDL ^c)^ | BDL ^c)^ |
| *M. globosa* ATCC MYA-4612 | 0 | 5.53 | 5.48 | 5.56 | 5.46 | 5.57 |
|  | 15 | n.t. ^b)^ | 4.22 | 3.50 | 2.70 | BDL ^c)^ |
|  | 30 | n.t. ^b)^ | 3.60 | 2.70 | BDL ^c)^ | BDL ^c)^ |
|  | 60 | 5.49 | 3.06 | 2.39 | BDL ^c)^ | BDL ^c)^ |

^a)^ Fungi was incubated for 15, 30 and 60 minutes with 0.25, 0.5, 1 or 2 mmol/L benzoyl peroxide (BPO). After incubation, the bacterial suspensions were collected and plated onto agar plates to calculate viable counts. Data indicate the mean of three repeated experiments. CFU: colony forming unit.

^b)^ Not tested.

^c)^ BDL: below the detection limit (<1.60 log CFU/mL).
